# Stunting in pre-school and school-age children in the Peruvian highlands and its association with *Fasciola* infection and demographic factors

**DOI:** 10.1371/journal.pntd.0009519

**Published:** 2021-06-21

**Authors:** Camille M. Webb, Maria Luisa Morales, Martha Lopez, Benicia Baca-Turpo, Eulogia Arque, A. Clinton White, Miguel M. Cabada

**Affiliations:** 1 Division of Infectious Diseases, Department of Internal Medicine, University of Texas Medical Branch, Galveston, Texas, United States of America; 2 Alexander von Humboldt Tropical Medicine Institute, Department of Medicine, Universidad Peruana Cayetano Heredia, Cusco, Peru; 3 UPCH–UTMB Collaborative Research Center—Cusco, Universidad Peruana Cayetano Heredia, Cusco, Peru; IRNASA, CSIC, SPAIN

## Abstract

Fascioliasis is a zoonotic trematode infection that is endemic in the highlands of Peru. Chronic fascioliasis can be asymptomatic and remain undiagnosed for years. Chronic malnutrition in children, as manifested by stunting, leads to delayed cognitive development and lost productivity. We hypothesized that fascioliasis is among the factors associated with stunting in children from endemic areas. We conducted a cross-sectional study among children attending pre-school and school in 26 communities in the Anta province in the Cusco region of Peru. We conducted interviews to collect information on demographic, socioeconomic, and medical history. Blood was collected and tested for complete cell count and FAS2 ELISA for *Fasciola* antibodies. Three stool samples per participant were tested for parasites by Kato-Katz and Lumbreras rapid sedimentation methods. Chronic fascioliasis was determined by the presence of ova in stool. Children’s height, weight, and age were recorded and used to calculate height for age Z scores (HAZ). Three thousand children participated in the study. Nine percent (264) of children had at least one positive test for *Fasciola* infection, 6% (164) had chronic fascioliasis, and 3% (102) had only positive antibody tests. The median HAZ was -1.41 (IQR: -2.03 to -0.81) and was similar in males and females. Twenty six percent (776) of children had stunting with HAZ < -2. Children with chronic fascioliasis had a lower median HAZ than children without *Fasciola* (-1.54 vs. -1.4, p = 0.014). History of treatment for malnutrition, history of treatment for anemia, having other helminths in stool, lower socioeconomic score, living at a higher elevation, and fewer years of schooling of both parents were associated with a lower HAZ score. In a multiple regression analysis, older age and a lower socioeconomic score were associated with a lower HAZ score. While fascioliasis and other helminths were associated with lower HAZ, they were not independent of the socioeconomic score.

## Author summary

*Fasciola* infection is endemic in the highlands of Peru. Children in the highlands are particularly affected. The effect of chronic fascioliasis on children’s long-term health and development, particularly the impact on nutrition, is not well defined. In this large epidemiologic study, we evaluated the association of chronic *Fasciola* infection with chronic malnutrition. Children with chronic *Fasciola*, defined as finding *Fasciola* eggs in stool, had a lower height for age Z score than those without *Fasciola*. A lower poverty score, fewer years of education of parents, and higher house elevation were also associated with stunted growth.

## Introduction

Fascioliasis, caused by the liver flukes *Fasciola hepatica* and *Fasciola gigantica*, is a zoonotic parasitic infection with worldwide distribution and high prevalence in some areas of South America, Africa, the Middle East, and Asia [1–3]. Fascioliasis is among the key diseases targeted by the WHO neglected tropical disease program [4]. Global estimates of infection range between 2.4 and 17 million people, and more than 180 million may be at risk. However, the true prevalence may be higher because most cases in the community remain undiagnosed. Few large epidemiologic studies have been conducted in endemic areas, using optimal methods to diagnose fascioliasis [3,5–10].

There are limited data on the health impacts of chronic *Fasciola* infection in humans, and the long-term nutritional effects of fascioliasis are not well characterized [11,12]. Fascioliasis can present as an acute infection during the parasites’ migration through the liver and as a chronic disease, which occurs once the adult flukes have established in the biliary tract [13,14]. Chronic *Fasciola* has been associated with anemia and weight loss. A study in Peru showed that children with chronic *Fasciola* infection were three times more likely to have anemia than uninfected children of similar age, gender, and other parasite infections [15]. Similarly, a study in Egypt showed that 62% of subjects diagnosed with chronic *Fasciola* infection had normocytic normochromic anemia [16]. A hospital-based case series from Lima in Peru reported weight loss in 48% of those presenting with acute infection and 37% of those with chronic infection [17].

Stunting is a marker of chronic malnutrition and is associated with developmental delays and followed by lost productivity and low wages in adulthood [18]. Interventions that target stunting have the potential to have an enormous impact on child development and human capital [19,20]. The cause of stunting is usually multifactorial, including dietary deficiencies, low socioeconomic status, and infections [21]. The association of intestinal helminth infections with malnutrition has led to global efforts to use mass chemotherapy for geohelminths. *Fasciola* infection may be a preventable factor related to chronic malnutrition and stunting, but few studies on this issue are available. We conducted a large community-based study among children in the Cusco region to determine the association between *Fasciola* infection and stunting, accounting for other factors that may influence malnutrition in this area.

## Methods

### Ethics statement

The study was approved by the institutional ethics committee of Universidad Peruana Cayetano Heredia in Peru and the Institutional Review Board of University of Texas Medical Branch in Galveston.

**Fig 1:**
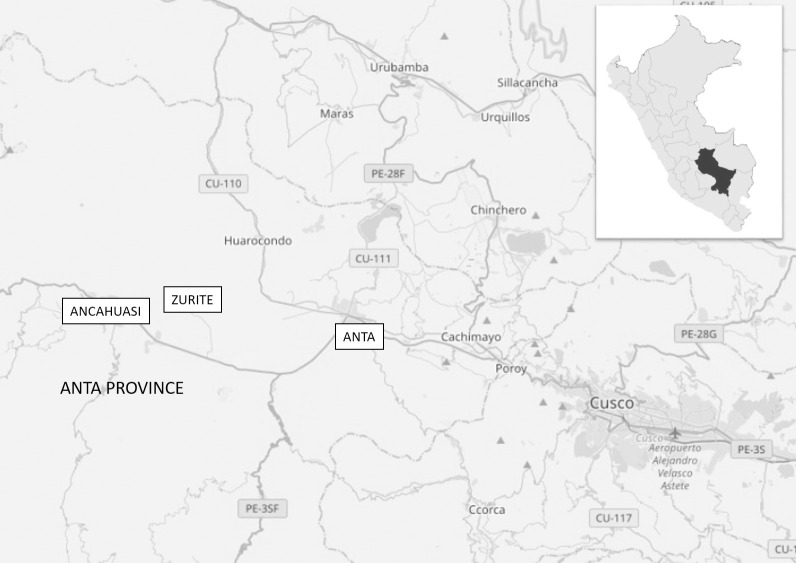
Map of the three districts included in the study (This map was created using Natural Earth http://www.naturalearthdata.com/about/terms-of-use/).

We performed a cross-sectional study to assess the association between chronic *Fasciola* infection and nutrition status among children in the Anta province of Cusco, Peru [22].[Fig 1] Between August 2013 and July 2018, we surveyed children ages 3 to 16 years attending pre-schools and schools in three districts of the Anta province of Cusco in Peru. Children were enrolled if they had never received *Fasciola* treatment, their parents gave signed informed consent (and assent for children older than five years). Interviews with children and their parents were conducted in schools to collect demographic, medical, and nutritional information, including anthropometric measurements. Interviews were followed by household visits to collect socioeconomic and environmental data. A school-based deworming program provided albendazole treatment twice a year to all children enrolled with a reported mean coverage over 90%.

Children’s weight and height were measured. Age was determined according to the reported date of birth [23]. The WHO Anthroplus software was used to calculate the height for age (HAZ) and weight for age (WAZ) Z scores [24]. Stunting and underweight were define following WHO Child Growth Standards [25].

The Simple Poverty Scorecard validated for Peru was used to estimate the probability of living under the 3.75/day United States Dollars (USD) poverty line among participating households [26]. This scorecard was validated in 2010 based on Peruvian government data and is designed to estimate the household’s ’poverty likelihood.’ The raw score was used as a continuous variable. The likelihood of ≥ 48.8% of living under the set poverty line (equivalent to a score of fewer than 34 points) was used as a dichotomous variable.

We calculated a Food Consumption Score (FCS) using a United Nations food questionnaire adapted to reflect local dietary habits [27]. The questionnaire groups common locally consumed items in food categories to create a scorecard to estimate food security (S1 Table). The score was calculated by multiplying each food group’s frequency of consumption by the food group’s assigned weight. Scores ranged between 0 and 112 points. Because this population had a high oil and sugar intake, cutoffs were adjusted from 21 to 28 and 35 to 42 points as recommended [28]. A score below 28 was considered severely food insecure, scores between 28 and 42 were considered moderately food insecure, and scores >42 points were considered food secure.

One blood sample and three consecutive stool samples were collected from each participant. Blood samples were tested for complete blood count using an automated method (BC-5300 Auto Hematology Analyzer, MindRay, Shenzhen, China). Hemoglobin values were adjusted for chronic high-altitude exposure using the Center for Disease Control and Prevention formula and the elevation of the participant’s house [29]. Anemia was defined using the WHO age and sex-adjusted hemoglobin cutoffs [30]. Antibodies to the Fas2 antigen of *F*. *hepatica* were tested using a commercial enzyme linked immunosorbent assay (Fas2 ELISA, Bionoma SRL, Lima, Peru) [31]. Each freshly collected stool sample was tested by the Kato Katz test within 48 hours of collection. Stool preserved in 10% formalin was tested with Lumbreras rapid sedimentation test within one week to evaluate for the presence of *Fasciola* and other helminth eggs or larvae [32]. A second observer examined all slides positive for helminths to confirm the results. All samples were processed at the UPCH-UTMB collaborative research center in Cusco.

The statistical analysis was performed using SAS Studio 5.2 (SAS Institute Inc., Cary, NC, USA). HAZ score as a continuous variable was used as the primary dependent variable. Stunting, defined as HAZ score less than two standard deviations below the mean, was used as a categorical dependent variable. Chronic fascioliasis was defined as having positive stool microscopy for *Fasciola* eggs. *Fasciola* infection was defined as having positive stool microscopy or having positive *Fasciola* Fas2 ELISA. We calculated medians and interquartile ranges for numeric variables and frequencies for categorical values.

The distribution of the HAZ score was evaluated using the Kolmogorov-Smirnov test. The Spearman’s R and Mann-Whitney U tests were used to evaluate the HAZ score association with the predictor variables. The association of HAZ with significant demographics, socioeconomic factors, food security score, and helminth infections was evaluated using a multivariable linear regression model.

A two-sided p-value <0.05 was used to define statistical significance. The 95% confidence interval was estimated for our main results. All subjects for whom there were data available were included in every analysis. When more than 10% of values were missing for a variable, the distribution of HAZ, stunting, chronic fascioliasis, and poverty score were compared between those with missing and no-missing values. If a significant difference was encountered, the analysis was conducted with and without the variable.

## Results

Three thousand children ages 3 to 16 years from 2124 households were included in the study. Participants’ median age was 9.6 (IQR 6.8–12.4) years, and half (50.1%) were female. Most children were from the Anta district (51.2%) and lived in households at a median elevation of 3390 (IQR 3342–3488) meters. Mothers and fathers had a median of 6 (IQR 6–11) and 8 (IQR 3–10) years of schooling, respectively. Thirty-eight percent (972/2581, 37.7%) had a greater than 48.8% likelihood of living under the USD 3.75/day poverty line. One hundred eleven (3.9%) children had been previously treated for malnutrition and 392 (13.6%) for parasite infections. The median food consumption score was 84 points (IQR 72–93). The household of most participants was considered food secure (FCS >42) (2941, 99.5%) (Table 1).

**Table 1:** Demographic, socioeconomic, and clinical characteristics of the participants.

Variable		N (%)/Median (IQR)
*Sex*	Male	1495 (49.9)
	Female	1503 (50.1)
Median age (years)		9.6 (6.8–12.4)
*District*	Ancahuasi	1150 (38.3)
	Anta	1537 (51.2)
	Zurite	313 (10.4)
*House construction material*	Adobe	2709 (91.8)
	Brick/ Other	241 (8.2)
Median Simple Poverty Scorecard		38 (31–45)
≥48.8% likelihood of living below 3.75 USD^‡^		972 (37.7)
*Food Security Group (Adjusted)*^*†*^	Food Secure	2941 (99.53)
	Moderately Food Insecure	13 (0.44)
	Severely Food Insecure	1 (0.03)
Previous treatment for anemia^§^		175 (6.1)
Previous treatment for malnutrition^§^		111 (3.9)
Previous treatment for parasites^§^		392 (13.6)
HAZ		-1.41 (-2.03, -0.81)
WAZ		-0.51 (-1.06, 0.07)
BMI		17.76 (15.96–18.87)
Stunting (HAZ< -2)^¶^		776 (26)
Hemoglobin corrected for altitude (g/dL)		12.7 (12.0–13.5)

‡ Socioeconomic score has a range of 0 to 100 points, with higher points related to a lower likelihood of living under 3.75 USD.

† Food Consumption Score is on a scale of 0 to 112, with a higher value related to food security. A cutoff of 42 points is used to consider being food secure.

§As recalled by parents at the time of the interview.

¶Using WHO age and sex-adjusted cutoff

The median HAZ score was -1.4 (IQR -2.03 to -0.81), and the median WAZ was -0.51 (-1.06 to -0.07). Stunting was present in 776/2986 participants (26.0%). The prevalence of anemia was 11.4% (340/2983) (Table 1).

Two hundred sixty-four subjects of 2998 (8.8%) had *Fasciola* infection. Of these, 164/2976 (5.5%) had chronic fascioliasis, 220/2989 (7.4%) had positive *Fasciola* serology, and 118 (3.9%) had both. Of participants with *Fasciola* eggs in stool, the geometric mean of egg count was 39.5 eggs per gram of stool, ranging from 6.7 to 820 eggs per gram of stool. *Giardia intestinalis* (23.5%) and *Hymenolepis nana* (16.3%) were the most common parasites found in stool testing. Twenty-seven percent of participants (806/2989) had at least one intestinal helminth other than *Fasciola*, and 127 (4.2%) had more than one intestinal helminth (S2 and S3 Tables).

Children with stunting were older than those without stunting (10.5 vs. 9.4 years, p < 0.01).

Older age, higher house elevation, lower socioeconomic score, and fewer years of schooling of both parents were associated with a lower HAZ score (p < 0.001). Children with a history of treatment for anemia (p = 0.02) or for malnutrition (p = 0.004) had a lower median HAZ score. Similarly, those with >48.8% likelihood of living under USD 3.75/day poverty line had a lower median HAZ score (p = 0.001). Participants from the Ancahuasi district had a lower HAZ score as compared to those from Anta and Zurite (-1.51 versus -1.36 and -1.44, p = 0.005).

Participants with at least one positive test for *Fasciola* had a lower median HAZ score than those not exposed to *Fasciola* (-1.54 vs. -1.4, p = 0.015), and those with chronic *Fasciola* also had a lower median HAZ score compared to those without *Fasciola* eggs in the stool (-1.57 vs. -1.4, p = 0.022). Positive serology for *Fasciola* alone was not associated with a lower HAZ score. The presence of other helminths in stool was associated with a lower median HAZ score (-1.47 vs. -1.39, p = 0.01) (Table 2). Participants with more than one intestinal helminth had a lower HAZ score than those with one gastrointestinal helminth infection (-1.65 vs. -1.44, p = 0.003).

**Table 2:** Bivariate analysis of the association between HAZ score and demographic, socioeconomic, and parasitological variables.

Variable	Categories	Median HAZ (IQR)	P-value
Sex	Male	-1.40 (-1.99 to -0.82)	0.45
	Female	-1.42 (-2.06 to -0.81)	
District	Ancahuasi	-1.51 (-2.15 to -0.82)	0.005
	Anta	-1.36 (-1.95 to -0.81)	
	Zurite	-1.44 (-1.92 to -0.84)	
>48% likelihood of living under 3.75USD per month	Yes	-1.68 (-2.23 to -1.01)	<0.001
	No	-1.28 (-1.88 to -0.68)	
Food Security Group (Adjusted)	Food Secure	-1.41 (-2.02 to -0.81)	0.26
	Food Insecure	-1.79 (-2.20 to -1.33)	
Previous treatment for anemia^§^	Yes	-1.60 (-2.12 to -1.00)	0.02
	No	-1.39 (-20 to -0.81)	
Previous treatment for malnutrition^§^	Yes	-1.73 (-2.29 to -1.04)	0.004
	No	-1.39 (-2.00 to -0.81)	
Previous treatment for parasites^§^	Yes	-1.52 (-2.00 to -0.88)	0.2
	No	-1.39 (-2.02 to -0.81)	
Anemia present	Yes	-1.55 (-2.11 to -1.04)	0.002
	No	-1.39 (-2.02 to -0.80)	
*Fasciola* in stools	Yes	-1.59 (-2.09 to -1.08)	0.02
	No	-1.4 (-2.01 to -0.81)	
Any positive *Fasciola* test	Yes	-1.52 (-2.10 to -1.08)	0.015
	No	-1.4 (-2.01 to -0.80)	
Positive *Fasciola* serology	Yes	-1.46 (-2.05 to -0.99)	0.27
	No	-1.40 (-2.02 to -0.81)	
Other helminths (not *Fasciola*)	Yes	-1.52 (-2.08 to -0.89)	0.022
	No	-1.37 (-2.01 to -0.80)	
Any helminth in stool	Yes	-1.52 (-2.08 to -0.90)	0.01
	No	-1.37 (-2.02 to -0.80)	
Any parasite in stool	Yes	-1.42 (-2.00 to -0.82)	0.97
	No	-1.39 (-2.04 to -0.80)	
Variable		Spearman R correlation coefficient	P-value
Age		-0.17	<0.001
House elevation (meters above sea level)		0.14	<0.001
Socioeconomic score (Poverty scorecard)		0.22	<0.001
Father’s years of schooling		0.18	<0.001
Mother’s years of schooling		0.25	<0.001
Food Consumption Score		0.06	<0.001
Corrected hemoglobin		0.004	0.81

§As recalled by parents at the time of the interview.

The socioeconomic score had 419 missing values (14%), and father’s years of schooling had 455 missing values (15.2%). All other variables had fewer than 5% missing values. The distribution of the HAZ score and *Fasciola* in stools was not different in those with missing values as compared to those without missing values. The socioeconomic score was not significantly different in those with missing father’s years of schooling.

There was a high correlation between the socioeconomic score with fathers’ years of education (r_s_ = 0.36, p < 0.001), mothers’ years of education (r_s_ = 0.48, p < 0.001), and house elevation (r_s_ = -0.22, p < 0.001). Due to the high correlation between these variables, only the socioeconomic score was included in the multivariate analysis to avoid redundant variables. In the multiple regression analysis, older age and lower socioeconomic score were significantly associated with a lower HAZ score. The model predicted 0.09% of the variance in the HAZ score (Table 3). *Fasciola* in stools and other helminth infections were not significant independent variables. Additional models, which included any *Fasciola* infection, positive *Fasciola* serology, intensity of infection (Kato Katz egg count), and number of parasites in stool, similarly did not show an independent association of *Fasciola* infection with lower HAZ score.

**Table 3:** Multiple regression analysis of factors associated with Height for Age Z score.

Variable	Unstandardized Coefficient	95% Confidence Interval	p value
Sex	-0.075	-0.15 to -0.002	0.044
Age	-0.045	-0.06 to -0.03	< .0001
District	-0.035	-0.03 to -0.10	0.742
Socioeconomic score	0.017	0.01 to 0.02	< .0001
Food score	0.001	0.00 to 0.003	0.566
Previous treatment for anemia	-0.196	-0.40 to 0.01	0.124
Previous treatment for malnutrition	-0.152	-0.41 to 0.10	0.170
Previous treatment for parasites	-0.060	-0.18 to 0.06	0.295
*Fasciola* in stool	-0.052	-0.21 to 0.11	0.432
Other helminths in stool	-0.006	-0.10 to 0.08	0.770

When looking at the Ancahuasi district, which had the highest prevalence of *Fasciola* infection, older age, lower socioeconomic score, and history of treatment for malnutrition, were associated with a lower HAZ score (S5 Table). The presence of *Fasciola* in stools or other helminths in stools was not independently associated with a lower HAZ score in the Ancahuasi district.

## Discussion

Few studies have evaluated the clinical implications of chronic *Fasciola* infection in human health. Although hospital case series and outbreak reports describe weight loss in infected subjects, the complex relationships of fascioliasis, other risk factors, and chronic malnutrition have not been studied at the community level. In this large epidemiologic study, children with chronic *Fasciola* had a lower HAZ score. Other factors associated with a lower HAZ score were lower socioeconomic scores, lower hemoglobin values, and living at higher elevation. Children from the Ancahuasi district had a lower HAZ score than those from other districts. Sex, age, and lower socioeconomic score were associated with HAZ in the multiple regression model. Despite low levels of food insecurity, there was a high prevalence of stunting among children. This underscores the importance of factors other than food in the development of malnutrition. The complex interplay between demographics, socioeconomics, helminth infections, and malnutrition was evident in our study.

Children with either chronic *Fasciola* or helminth infections had a lower HAZ score than those without infections in the bivariate analysis, though these infections did not independently predict a lower HAZ score in the multivariate analysis. The effect of chronic *Fasciola* infection is difficult to interpret due to the multifaceted nature of stunted growth. Several other factors may contribute to the lack of predictive value of these infections in our model of HAZ score. *Fasciola*, as well as other helminth infections, had a low egg burden in our population, as demonstrated by low geometric means of egg counts. The prevalence of *Fasciola* infection in our study was 8.8%, with a total of 164 participants with chronic fascioliasis. Thus, the study may have been underpowered to assess this association in a multivariate model. Prior studies in this area show similar prevalence, though with high variation among communities. Parkinson et al [3] described an overall infection level of 18.5% in the Bolivian altiplano, with rates that varied from <10% to 53%. Mas-Coma has described rates of infection of 15.6% in the Puno region, and rates as high as 72% and 100% have been reported in Bolivia [33]. In our study, older children had a higher prevalence of *Fasciola* infection [34]. The distribution of *Fasciola* infection among age groups varies among study regions [35]. Further understanding of the time at which children are more at risk of *Fasciola* infection may help clarify the impact of fascioliasis on nutrition and development. Prior studies from our group in the Cusco Region describe the association between *Fasciola* infection and the likelihood of living in poverty, fewer years of parents’ education, and house elevation [15,22]. Children living in poverty may have increased environmental exposure to *Fasciola* and other parasitic infections. Fewer years of parents’ education has also been associated with an increased risk of parasitic infections in children [36]. The same factors may predispose children to inadequate nutrition and stunted growth through different mechanisms. Poor maternal health and nutrition, recurrent infections, and adolescent pregnancy, are among the factors that contribute to stunted growth. Subclinical infections, particularly during the first years of life, have been linked to stunting through chronic inflammation [37].

Stunting was associated with living at a higher elevation in our study population. In Peru, stunting and anemia among children are more common in rural areas and in the highlands. In 2017, stunting in rural areas was three times higher than in urban areas. Similarly, stunting in the highlands was three times higher than in the coast, but other low land areas in the jungle showed smaller differences [38]. Higher poverty levels and lower years of education of the parents; two factors associated with stunting in children; follow the same rural and elevation patterns in Peru [39]. In our study, a contributory factor may be that children living at a higher altitude had higher rates of *Fasciola* infection. The *Galba truncatula* snails involved in *Fasciola* transmission in the highlands of South America produce more cercariae, for a longer period, and live longer than snails at lower altitudes, likely increasing the risk of infection in those environments [40].

Parasite burden of soil transmitted helminths in our study was low, with prevalence of *Ascaris* of 4.9%, hookworm of 0.9%, and *Trichuris* of 0.6%. Helminth infections during early childhood have been associated with stunted growth, and there is geographic overlap between areas with a high burden of helminths and areas with childhood malnutrition. In a study in rural Kenya, parasitic infections during the first three years of life, particularly hookworm, *Ascaris*, *E*. *histolytica*, malaria, and *Schistosoma* infection, were associated with decreased growth [41]. In a study of 244 children under three years of age in rural Kenya, child parasitic infectious disease burden was associated with stunted growth, though this was primarily driven by malaria infection during the first three years of life [42]. The odds ratio of stunted growth increased by 1.41 with each parasitic infection during childhood (95% CI 1.05, 1.95). A study that looked at the interaction of helminth infections and nutrition in school-aged children in the northern Philippines found that having one or more parasitic infections was associated with lower caloric intake [43]. These studies have been primarily in children younger than our study population. The cross-sectional nature of these studies makes it difficult to evaluate the causality of these associations fully.

One in four children in our study population had stunting. A higher poverty score, fewer years of education of parents, and higher home elevation were all associated with lower HAZ score. These socioeconomic determinants have been associated to stunting in Peru and in other developing countries. The country prevalence in children less than five years old, as described in the 2017 ENDES survey, was 12.9%. However, in the highlands, the prevalence was 21.3%, with some districts in the highlands reaching rates of 32%. Rates of malnutrition in Peru are higher in rural areas (25.7%) as compared to urban areas (7.3%). Stunting in Peru is highest in children living in the lowest poverty quintile [38]. The association of malnutrition with fewer years of education of parents has also been previously described. In a study in Mozambique, Ghana, and Nigeria, children of parents with higher years of education were more likely to have adequate growth [44]. A study of stunting determinants in the Democratic Republic of Congo found an association of fewer years of education of mothers with stunting and access to safe water, sewage, and mother’s BMI.

Our study has several limitations. The cross-sectional design did not detect prior episodes of fascioliasis, which may have resolved or have been treated before the current study but may have affected linear growth. The Fas2 ELISA test does not accurately differentiate between past and current infections, and some participants in the study with a positive Fas2 ELISA and negative eggs in stool may have had fascioliasis [45]. Other factors associated with malnutrition, such as birth weight, age of parents, and breastfeeding, were not measured [21,46] though these are particularly important in younger children. The inclusion of the poverty scorecard in the multivariate analysis helps account for possible differences in socioeconomic development. Despite these limitations, our study has several strengths. We evaluated and interviewed 3000 children and conducted thorough evaluations of socioeconomic factors and demographics, anthropometric measures, laboratory values, and data on food security. We used more than one stool sample per child and serology testing to increase our sensitivity of detecting *Fasciola* infection.

Poverty has been associated with a higher prevalence of infections, stunting in growth, and delays in development. Deworming programs have been widely implemented globally due to the effects of helminth infections on growth and cognitive development. *Fasciola* has not been included in usual deworming programs as it does not respond to common anti-helminthic drugs used in deworming programs. Its control is difficult due to the many hosts involve in its lifecycle, underdiagnosis, and decreasing effectiveness of triclabendazole, the only WHO recommended drug to treat fascioliasis. Our study adds to the evidence of a complex relationship between parasite infections, nutrition, and socioeconomic factors in this area.

## Supporting information

S1 Table Groups of items in each food group. (DOCX)

S2 Table Prevalence of parasite infections. (DOCX)

S3 Table Geometric mean of parasite infections. (DOCX)

S4 Table Backwards logistic regression analysis of variables associated with stunting. (DOCX)

S5 Table Multiple regression analysis of factors associated with Height for Age Z score in Ancahuasi district. (DOCX)

## References

[1] Heydarian P, Ashrafi K, Mohebali M, Kia EB, Aryaeipour M, Chegeni Sharafi A, et al. Seroprevalence of Human Fasciolosis in Lorestan Province, Western Iran, in 2015–16. Iran J Parasitol. 2017;12(3):389–97. 28979349 PMC5623919

[2] Carmona C, Tort JF. Fasciolosis in South America: epidemiology and control challenges. J Helminthol. 2017;91(2):99–109. doi: 10.1017/S0022149X16000560 27608827

[3] Parkinson M, O’Neill SM, Dalton JP. Endemic human fasciolosis in the Bolivian Altiplano. Epidemiol Infect. 2007;135(4):669–74. doi: 10.1017/S095026880600728X 17064455 PMC2870614

[4] WHO. Control of Neglected Tropical Diseases. 2020.

[5] Furst T, Keiser J, Utzinger J. Global burden of human food-borne trematodiasis: a systematic review and meta-analysis. Lancet Infect Dis. 2012;12(3):210–21. doi: 10.1016/S1473-3099(11)70294-8 22108757

[6] Nguyen TG, Le TH, De NV, Doan TT, Dao TH, Vercruysse J, et al. Assessment of a 27-kDa antigen in enzyme-linked immunosorbent assay for the diagnosis of fasciolosis in Vietnamese patients. Trop Med Int Health. 2010;15(4):462–7. doi: 10.1111/j.1365-3156.2010.02468.x 20149166

[7] Curtale F, Hassanein YA, Barduagni P, Yousef MM, Wakeel AE, Hallaj Z, et al. Human fascioliasis infection: gender differences within school-age children from endemic areas of the Nile Delta, Egypt. Trans R Soc Trop Med Hyg. 2007;101(2):155–60. doi: 10.1016/j.trstmh.2006.05.006 16890257

[8] Haseeb AN, el-Shazly AM, Arafa MA, Morsy AT. A review on fascioliasis in Egypt. J Egypt Soc Parasitol. 2002;32(1):317–54. 12049266

[9] Curtale F, Hassanein YA, Savioli L. Control of human fascioliasis by selective chemotherapy: design, cost and effect of the first public health, school-based intervention implemented in endemic areas of the Nile Delta, Egypt. Trans R Soc Trop Med Hyg. 2005;99(8):599–609. doi: 10.1016/j.trstmh.2005.03.004 15935413

[10] Rokni MB, Massoud J, O’Neill SM, Parkinson M, Dalton JP. Diagnosis of human fasciolosis in the Gilan province of Northern Iran: application of cathepsin L-ELISA. Diagn Microbiol Infect Dis. 2002;44(2):175–9. doi: 10.1016/s0732-8893(02)00431-5 12458125

[11] Hotez PJ, Bottazzi ME, Franco-Paredes C, Ault SK, Periago MR. The neglected tropical diseases of Latin America and the Caribbean: a review of disease burden and distribution and a roadmap for control and elimination. PLoS Negl Trop Dis. 2008;2(9):e300. doi: 10.1371/journal.pntd.0000300 18820747 PMC2553488

[12] Webb CM, Cabada MM. Recent developments in the epidemiology, diagnosis, and treatment of Fasciola infection. Curr Opin Infect Dis. 2018;31(5):409–14. doi: 10.1097/QCO.0000000000000482 30113327

[13] Cabada MM, White AC Jr. New developments in epidemiology, diagnosis, and treatment of fascioliasis. Curr Opin Infect Dis. 2012;25(5):518–22. doi: 10.1097/QCO.0b013e3283567b7e 22744320

[14] Moazeni M, Ahmadi A. Controversial aspects of the life cycle of *Fasciola hepatica*. Exp Parasitol. 2016;169:81–9. doi: 10.1016/j.exppara.2016.07.010 27475124

[15] Lopez M, White AC Jr., Cabada MM. Burden of *Fasciola hepatica* Infection among children from Paucartambo in Cusco, Peru. Am J Trop Med Hyg. 2012;86(3):481–5. doi: 10.4269/ajtmh.2012.11-0448 22403322 PMC3284367

[16] El-Shazly AM, El-Nahas HA, Abdel-Mageed AA, El Beshbishi SN, Azab MS, Abou El Hasan M, et al. Human fascioliasis and anaemia in Dakahlia Governorate, Egypt. J Egypt Soc Parasitol. 2005;35(2):421–32. 16083056

[17] Chang Wong MR, Pinto Elera JOA, Guzman Rojas P, Terashima Iwashita A, Samalvides Cuba F. Demographic and clinical aspects of hepatic fascioliasis between 2013–2010 in National Hospital Cayetano Heredia, Lima, Peru. 2016. 27131937

[18] WHO. Nutrition [August 30, 2020]. Available from: https://www.who.int/nutrition/en/.

[19] Perkins JM, Kim R, Krishna A, McGovern M, Aguayo VM, Subramanian SV. Understanding the association between stunting and child development in low- and middle-income countries: Next steps for research and intervention. Soc Sci Med. 2017;193:101–9. doi: 10.1016/j.socscimed.2017.09.039 29028557

[20] Bouma S. Diagnosing Pediatric Malnutrition. Nutr Clin Pract. 2017;32(1):52–67. 10.1177/0884533616671861 27765878

[21] de Onis M, Branca F. Childhood stunting: a global perspective. Matern Child Nutr. 2016;12 Suppl 1:12–26. doi: 10.1111/mcn.12231 27187907 PMC5084763

[22] Cabada MM, Morales ML, Webb CM, Yang L, Bravenec CA, Lopez M, et al. Socioeconomic Factors Associated with *Fasciola hepatica* Infection Among Children from 26 Communities of the Cusco Region of Peru. Am J Trop Med Hyg. 2018;99(5):1180–5. doi: 10.4269/ajtmh.18-0372 30226136 PMC6221222

[23] WHO. Child Growth Standards: Measuring a Child’s Growth. 2008.

[24] WHO | Application tools Anthroplus Software. WHO. 2018.

[25] WHO. WHO | The WHO Child Growth Standards. WHO. 2016.

[26] Schreiner M. A Simple Poverty Scorecard for Peru 2012 [Available from: http://microfinance.com/English/Papers/Scoring_Poverty_Peru_2010_EN.pdf.

[27] Programme WF. Meta Data for the Food Consumption Score (FCS) Indicator. 2015.

[28] Technical Guidance Sheet—Food Consumption Analysis: Calculation and Use of the Food Consumption Score in Food Security Analysis | WFP | United Nations World Food Programme—Fighting Hunger Worldwide 2018 [Available from: https://www.wfp.org/content/technical-guidance-sheet-food-consumption-analysis-calculation-and-use-food-consumption-score-food-s.

[29] Nestel P. Adjusting hemoglobin values in program surveys. Washington, DC, USA.: International Nutritional Anemia Consultative Group, USAID; 2002 [Available from: https://pdf.usaid.gov/pdf_docs/PNACQ927.pdf.

[30] WHO. Haemoglobin concentrations for the diagnosis of anaemia and assessment of severity.

[31] Espinoza J, Maco V, Marcos L, Saez S, Neyra V, Terashima A, et al. Evaluation of Fas2-ELISA for the serological detection of *Fasciola hepatica* infection in humans. Am J Trop Med Hyg. 2007;76(5). 17488926

[32] Lopez M, Morales M, Konana M, Hoyer P, Pineda-Reyes R, White A, et al. Kato-Katz and Lumbreras Rapid Sedimentation Test to Evaluate Helminth Prevalence in the Setting of a School-Based Deworming Program. Pathog Glob Health. 2016;110(3):130–4. doi: 10.1080/20477724.2016.1187361 27376503 PMC4984960

[33] Mas-Coma S, Angles R, Esteban JG, Bargues MD, Buchon P, Franken M, et al. The Northern Bolivian Altiplano: a region highly endemic for human fascioliasis. Trop Med Int Health. 1999;4(6):454–67. doi: 10.1046/j.1365-3156.1999.00418.x 10444322

[34] Esteban JG, Flores A, Angles R, Mas-Coma S. High endemicity of human fascioliasis between Lake Titicaca and La Paz valley, Bolivia. Trans R Soc Trop Med Hyg. 1999;93(2):151–6. doi: 10.1016/s0035-9203(99)90289-4 10450437

[35] Mas-Coma MS, Esteban JG, Bargues MD. Epidemiology of human fascioliasis: a review and proposed new classification. Bull World Health Organ. 1999;77(4):340–6. 10327713 PMC2557647

[36] Quihui L, Valencia ME, Crompton DW, Phillips S, Hagan P, Morales G, et al. Role of the employment status and education of mothers in the prevalence of intestinal parasitic infections in Mexican rural schoolchildren. BMC Public Health. 2006;6:225. doi: 10.1186/1471-2458-6-225 16956417 PMC1584408

[37] Prendergast AJ, Rukobo S, Chasekwa B, Mutasa K, Ntozini R, Mbuya MN, et al. Stunting is characterized by chronic inflammation in Zimbabwean infants. PLoS One. 2014;9(2):e86928. doi: 10.1371/journal.pone.0086928 24558364 PMC3928146

[38] National Center for Food and Nutrition, National Institute of Health (Peru), National Institute of Statistics and Informatics (Peru), National Police of Peru (PNP). Peru Demographic and Family Health Survey 2017 Lima, Peru: National Institute of Statistics and Informatics (Peru). 2017. Available from: http://ghdx.healthdata.org/record/peru-demographic-and-family-health-survey-2017.

[39] National Institute of Statistics and Informatics (Peru). Peru National Household Survey 2019. Lima, Peru: National Institute of Statistics and Informatics (Peru). 2019. Available from: https://www.inei.gob.pe/media/DATOS_ABIERTOS/ENAHO/DICCIONARIO/2019/Anual/Diccionario.pdf.

[40] Vignoles P, Favennec L, Dreyfuss G, Rondelaud D. Highland populations of *Lymnaea truncatula* infected with *Fasciola hepatica* survive longer under experimental conditions than lowland ones. Parasitol Res. 2002;88(4):386–8. doi: 10.1007/s00436-001-0542-y 11999030

[41] LaBeaud AD, Nayakwadi Singer M, McKibben M, Mungai P, Muchiri EM, McKibben E, et al. Parasitism in Children Aged Three Years and Under: Relationship Between Infection and Growth in Rural Coastal Kenya. PLoS Negl Trop Dis. 2015;9(5):e0003721 doi: 10.1371/journal.pntd.0003721 25996157 PMC4440755

[42] Martin S, Mutuku F, Sessions J, Lee J, Mukoko D, Malhotra I, et al. Factors Associated With Early Childhood Stunted Growth in a 2012–2015 Birth Cohort Monitored in the Rural Msambweni Area of Coastal Kenya: A Cross-Sectional Study. BMC pediatrics. 2020;20(1):208. doi: 10.1186/s12887-020-02110-z 32398049 PMC7216696

[43] Papier K, Williams GM, Luceres-Catubig R, Ahmed F, Olveda RM, McManus DP, et al. Childhood Malnutrition and Parasitic Helminth Interactions. Clin Infect Dis. 2014;59(2):234–43. doi: 10.1093/cid/ciu211 24704723

[44] Amugsi DA, Dimbuene ZT, Kimani-Murage EW. Socio-demographic Factors Associated With Normal Linear Growth Among Pre-School Children Living in Better-Off Households: A Multi-Country Analysis of Nationally Representative Data. PloS one. 2020;15(3):e0224118. doi: 10.1371/journal.pone.0224118 32160190 PMC7065827

[45] Sarkari B, Khabisi SA. Immunodiagnosis of Human Fascioliasis: An Update of Concepts and Performances of the Serological Assays. J Clin Diagn Res. 2017;11(6):Oe05–oe10. doi: 10.7860/JCDR/2017/26066.10086 28764235 PMC5535427

[46] Khan MN, Islam MM. Effect of exclusive breastfeeding on selected adverse health and nutritional outcomes: a nationally representative study. BMC Public Health. 2017;17(1):889. doi: 10.1186/s12889-017-4913-4 29162064 PMC5697409

