# Supplementary material for: Stunting in pre-school and school-age children in the Peruvian highlands and its association with Fasciola infection and demographic factors
Source: PLoS Negl Trop Dis. 2021 Jun 21;15(6):e0009519. doi: 10.1371/journal.pntd.0009519 (PMC8248620; doi:10.1371/journal.pntd.0009519)
Supplement: S1 Table — (DOCX) [file pntd.0009519.s001.docx]

S1 Table: Groups of items in each food group

| - Group | - Food group | - Weight |
| --- | --- | --- |
| - Group 1: Cereals/tubers | - Maiz (corn), Arroz (rice), pan (bread), tuberculos (root vegetables), mashua | - 2 |
| - Group 2: Pulses | - Quinua (quinoa), Nueces (nuts) | - 3 |
| - Group 3: Vegetables | - Vegetales (vegetables) | - 1 |
| - Group 4: Fruit | - Frutas (fruit) | - 1 |
| - Group 5: Animal protein | - Pescado (fish), pescado seco (dried fish), Carne Roja (red meat), Carne blanca (white meat), huevos (eggs) | - 4 |
| - Group 6: Dairy | - Leche (milk) | - 4 |
| - Group 7: Sugar | - Dulces (sweets) | - 0.5 |
| - Group 8: Oil | - Aceite (oil) | - 0.5 |
