# Supplementary material for: Stunting in pre-school and school-age children in the Peruvian highlands and its association with Fasciola infection and demographic factors
Source: PLoS Negl Trop Dis. 2021 Jun 21;15(6):e0009519. doi: 10.1371/journal.pntd.0009519 (PMC8248620; doi:10.1371/journal.pntd.0009519)
Supplement: S2 Table — (DOCX) [file pntd.0009519.s002.docx]

S2 Table: Prevalence of parasite infections

| Test description | N (%) |
| --- | --- |
| Stool tests (Rapid sedimentation and Kato Katz) |  |
| *B. hominis* | 1015 (33.8) |
| *Giardia intestinalis* | 705 (23.5) |
| *Hymenolepis nana* | 490 (16.3) |
| *Fasciola hepatica* | 164 (5.5) |
| *Ascaris lumbricoides* | 147 (4.9) |
| *Enterobius vermicularis* | 51 (1.7) |
| *Trichuris trichiura* | 27 (0.9) |
| Hookworm *(A. duodenale/ N. americanus)* | 19 (0.6) |
| *H. diminuta* | 6 (0.2) |
| Any parasite in stools | 1908 (63.6) |
| Any intestinal helminth | 806 (26.9) |
| Multiple intestinal helminths | 127 (4.2) |
| *Fasciola* serology (FAS 2 ELISA) | 220 (7.4) |
| Any positive test for *Fasciola* | 264 (8.8) |
