# Supplementary material for: Stunting in pre-school and school-age children in the Peruvian highlands and its association with Fasciola infection and demographic factors
Source: PLoS Negl Trop Dis. 2021 Jun 21;15(6):e0009519. doi: 10.1371/journal.pntd.0009519 (PMC8248620; doi:10.1371/journal.pntd.0009519)
Supplement: S3 Table — (DOCX) [file pntd.0009519.s003.docx]

S3 Table 3: Geometric mean of parasite infections

| Parasite | Prevalence |  | | Mean (±SD) | Geometric mean | | Range |
| --- | --- | --- | --- | --- | --- | --- | --- |
|  | n (%) |  | | Eggs/gram of stool | | | |
| *Fasciola hepatica* | 164 (5.5) |  | | 57.73 (± 75.7) | 39.95 | | 6.7 – 820 |
| *Hymenolepis nana* | 490 (16.3) |  | | 535.60 (± 1035.1) | 145.1 | | 6.7 – 8213 |
| *Ascaris lumbricoides* | 147 (4.9) |  | | 384.21 (± 685.1) | 158.7 | | 6.7 – 4333 |
| *Strongyloides stercoralis* | 36 (1.2) |  | | NA | NA | | NA |
| *Trichuris trichura* | 27 (0.9) |  | | 24.17 (± 19.3) | 18.5 | | 6.7 – 67 |
| Hookworm | 19 (0.6) |  | | 10.0 (± 4.7) | 9.4 | | 6.6 – 13 |
| Taenia sp | 9 (0.3) |  | | 411.43 (± 656.7) | 84.6 | | 6.7 – 1787 |
| Geometric mean was calculated from mean egg count for each participant, including only samples with positive counts. | | | | | | | |
|  | | |  | | |  |  |
