# Supplementary material for: Stunting in pre-school and school-age children in the Peruvian highlands and its association with Fasciola infection and demographic factors
Source: PLoS Negl Trop Dis. 2021 Jun 21;15(6):e0009519. doi: 10.1371/journal.pntd.0009519 (PMC8248620; doi:10.1371/journal.pntd.0009519)
Supplement: S4 Table — (DOCX) [file pntd.0009519.s004.docx]

S4 Table: Backwards logistic regression analysis of variables associated with stunting

| Parameter | Coefficient | Standard Error | T value |
| --- | --- | --- | --- |
| Age | -0.013 | 0.006 | 5.40 |
| Socioeconomic score | 0.006 | 0.002 | 7.99 |
| District | 0.046 | 0.019 | 3.14 |
| History of treatment for nutrition | -0.138 | 0.078 | -3.04 |
| Root MSE 0.428, R^2^ 0.0522, Adjusted R^2^ 0.0507 | | | |
| Sex, age, district of residence, socioeconomic score, food score, reported treatment for anemia, reported treatment for malnutrition, reported treatment for parasite infections, *Fasciola* in stools, other helminth infections were included in the analysis. | | | |
| In the backward regression analysis using stunting as the dependent variable, older age, lower socioeconomic score, and history of being treated for malnutrition were associated with a lower HAZ score. Chronic *Fasciola* infection, infection with other helminths, district of residence, and history of treatment for malnutrition were not retained by the model. | | | |

|  |  |
| --- | --- |
