# Supplementary material for: Stunting in pre-school and school-age children in the Peruvian highlands and its association with Fasciola infection and demographic factors
Source: PLoS Negl Trop Dis. 2021 Jun 21;15(6):e0009519. doi: 10.1371/journal.pntd.0009519 (PMC8248620; doi:10.1371/journal.pntd.0009519)
Supplement: S5 Table — (DOCX) [file pntd.0009519.s005.docx]

S5 Table: Multiple regression analysis of factors associated with Height for Age Z score in Ancahuasi district

| Variable | Unstandardized Coefficient | 95% Confidence Interval | p value |
| --- | --- | --- | --- |
| Sex | -0.068 | -0.19 to 0.05 | 0.27 |
| Age | -0.074 | 0.09 to -0.06 | <.0001 |
| Socioeconomic score | 0.015 | 0.01 to 0.02 | <.0001 |
| Food score | 0.000 | -0.002 to 0.004 | 0.88 |
| Previous treatment for anemia | 0.427 | -0.05 to 0.90 | 0.08 |
| Previous treatment for malnutrition | -0.777 | -1.30 to -0.26 | 0.00 |
| Previous treatment for parasites | -0.167 | -0.37 to 0.04 | 0.11 |
| *Fasciola* in stool | 0.002 | -0.24 to 0.24 | 0.98 |
| Other helminths in stool | -0.020 | -0.16 to 0.12 | 0.78 |
